# Insight into the role of p62 in the cisplatin resistant mechanisms of ovarian cancer

**DOI:** 10.1186/s12935-020-01196-w

**Published:** 2020-04-16

**Authors:** Xiao-Yu Yan, Xian-Zhi Qu, Long Xu, Si-Hang Yu, Rui Tian, Xin-Ru Zhong, Lian-Kun Sun, Jing Su

**Affiliations:** 1 Department of Pathophysiology, Key Laboratory of Pathobiology, Ministry of Education, College of Basic Medical Sciences, Jilin University, 126 Xinmin Street, Changchun, 130021 China; 2 Department of Hepatobiliary & Pancreatic Surgery, The Second Hospital of Jilin University, Jilin University, Changchun, 130021 Jilin China

**Keywords:** p62, Cisplatin, Ovarian cancer, Drug resistance, Autophagy

## Abstract

Cisplatin is a platinum-based first-line drug for treating ovarian cancer. However, chemotherapy tolerance has limited the efficacy of cisplatin for ovarian cancer patients. Research has demonstrated that cisplatin causes changes in cell survival and death signaling pathways through its interaction with macromolecules and organelles, which indicates that investigation into the DNA off-target effects of cisplatin may provide critical insights into the mechanisms underlying drug resistance. The multifunctional protein p62 works as a signaling hub in the regulation of pro-survival transcriptional factors NF-κB and Nrf2 and connects autophagy and apoptotic signals, which play important roles in maintaining cell homeostasis. In this review, we discuss the role of p62 in cisplatin resistance by exploring p62-associated signaling pathways based on current studies and our work. Insights into these resistance mechanisms may lead to more effective therapeutic strategies for ovarian cancer by targeting p62.

## Background

Ovarian cancer is a gynecologic cancer with a high mortality rate. In developed countries, the mortality rate of ovarian cancer is three times that of breast cancer and the 5-year survival rate of patients with stage IV ovarian cancer is only 28% [1]. In 1965, the platinum-containing drug cisplatin (*cis*-diamminedichloroplatinum) was found to exhibit antimicrobial activity, and subsequent studies demonstrated that platinum compounds have strong antitumor activity [2, 3]. In the 1980s, the first-line drugs for ovarian cancer were *cisplatin* and *cyclophosphamide*. *Carboplatin*, a second-generation platinum drug, showed equivalent therapeutic effects as cisplatin but with fewer toxic side effects [4, 5]. At present, the standard treatment for patients with advanced ovarian cancer is surgery, followed by six cycles of *paclitaxel* and *carboplatin* neoadjuvant chemotherapy [6]. However, most ovarian cancer patients relapse after treatment and eventually show no sensitivity to platinum drugs. Although several clinical trials have been conducted in recent years to improve the efficacy of platinum-based therapies (Table 1), chemotherapy resistance to platinum drugs remains an obstacle that limits the clinical application and efficacy of these drugs.

**Table 1:** Clinical trials for platinum-based chemotherapy in ovarian cancer

Characteristic	Cancer	Treatment regimen	Patients enrolled	PFS(mo)	OS(mo)
Stage III and Stage IV [7]	Ovarian	*Cisplatin* (75 mg per square meter of body-surface area) *Cyclophosphamide* (750 mg per square meter)	202	13	24
		*Cisplatin* (75 mg per square meter of body-surface area) *Paclitaxel* (135 mg per square meter over a period of 24 h).	184	18	38
Stage III and Stage IV [8] (NCT02655016)	Ovarian	*Niraparib* once daily after a response to platinum-based chemotherapy.	487	13.9	84% (24-month interim analysis)
		*Placebo* group once daily after a response to platinum-based chemotherapy	246	8.2	77% (24-month interim analysis)
Relapsed > 6 months following completion of platinum-based therapy [9] (NCT00083122)	Ovarian and Primary Peritoneal Carcinoma	*Cisplatin* (60 mg/m2 IV) *Flavopiridol* (100 mg/m2 IV, 24 h infusion; 21 day cycles)	40	4.3	16.1

**Fig. 1:**
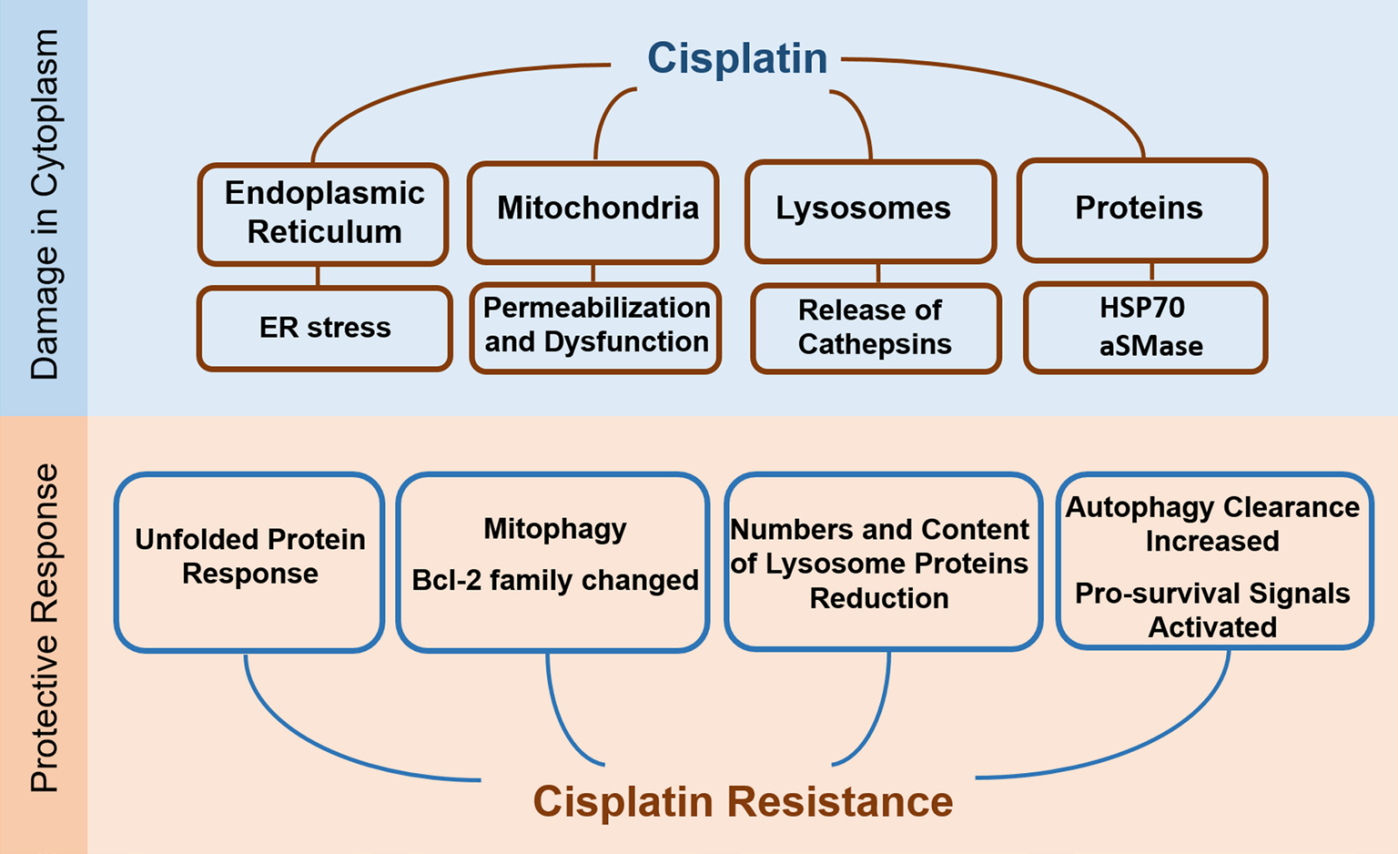
Cytoplasm effects induced by cisplatin in ovarian cancer cells. Cisplatin interacts with mitochondria, lysosomes, endoplasmic reticulum and cytoplasmic proteins, leading to cell stress and the activation of both death and pro-survival signals in ovarian cancer cells

Platinum is an electrophilic reagent characterized by its ability to form covalent linkages with nucleophilic residues of nucleobases such as guanine and adenine. Because a variety of cellular macromolecules contain nucleophilic residues, platinum drugs have the potential to interact with various cellular components, such as ribosomes, spliceosomes, and the RNA in telomerase, as well as proteins through Met, His and free Cys side chains [10, 11]. Galluzzi et al. [12] proposed that cisplatin can accumulate in mitochondria, lysosomes, endoplasmic reticulum, nucleus, cell membrane, cytoskeleton and cytosol, which causes cell stress. These findings indicate that cisplatin may exhibit far more effects on tumor cells than only through its interaction with DNA. And it may not only induce death signals, but also adaptive response including autophagy, the unfolded protein response and other pro-survival signals while disturbing organelles and proteins in the cytoplasm [13–15] (Fig. 1).

Although the signaling networks that determine cell survival and death are extensive, only a small number of molecules have been identified that function in coordinating these signaling pathways. The multifunctional protein p62/SQSTM1 (also known as sequestosome-1, hereinafter referred to as p62) integrates both survival and death signaling by regulating the ubiquitination of key cell signaling molecules that control survival and death [16–19]. p62 contains multiple protein-binding domains: the N-terminal PB1 (Phox and Bem1p) domain that binds to the atypical kinase (aPKC) and mediates p62 self-oligomerization; the central zinc finger (ZZ) domain that promotes NF-κB pathway activation; a TB module (motif) that binds TNF receptor associated factor 6 (TRAF6); the KIR (Keap1-interacting region) domain that competes with NRF2 for Keap1; a UBA (ubiquitin-associated) domain that recruits ubiquitin-linked proteins and mediates their degradation through autophagy or the ubiquitin–proteasome system; and the LIR (LC3-interacting region), which recognizes a specific sequence in the autophagosome membrane protein LC3. These multiple domains make p62 an important player in the regulation of selective autophagy [20, 21].

In this review, we discuss the changes in p62-mediated signaling pathways in ovarian cancer during cisplatin treatment based on our work and current research. We describe a role for p62 in cisplatin resistance of ovarian cancer, providing a theoretical basis for potential strategies for overcoming chemotherapy resistance in ovarian cancer.

### p62 participates in drug resistance of ovarian cancer by regulating autophagy

#### Macroautophagy

During macroautophagy, hereinafter referred to as autophagy, autophagosomes isolate components targeted for autophagy by forming a closed membrane structure and transporting them to lysosomes for degradation. Autophagy serves a protective function against malignant transformation and maintains homeostasis in normal tissues. However, once cells undergo transformation, autophagy provides cancer-protective functions to deal with stress from the worse survival environment [22, 23]. Previous reports demonstrated that cisplatin activates autophagy through the MEK/ERK pathway in ovarian cancer, which may lead to cisplatin resistance [24].

p62 is an autophagy receptor involved in the recognition of ubiquitin-labeled substrates targeted for autophagy [25]. Matsumoto et al. [26] suggested that p62-mediated selective autophagy is a compensatory pathway for protease degradation. The UBA domain structure in p62 forms a compact triple-spiral stalk with a hydrophobic surface, which may be a targeted contact site for protein interactions [27, 28]. Additionally, p62 binds the autophagy membrane protein Atg8/LC3 through the LIR. Current studies suggest D335, D336, D337, and W338 mutations eliminate the binding of p62 to LC3 [29]. We previously found that the levels of mature LC3II were increased in cisplatin-resistant SKOV3/DDP ovarian cancer cells compared with parental cells [30]. When we suppressed autophagy in ovarian cancer cells with 3-MA or chloroquine, cisplatin showed increased efficacy; furthermore, p62 expression was increased in SKOV3/DDP cells. Upon cisplatin treatment, p62 and LC3 puncta were co-localized. RNAi-mediated downregulation of p62 also increased the sensitivity of ovarian cancer cells to cisplatin [31]. These results indicate that p62-mediated autophagy induced by cisplatin may function as a protective mechanism in ovarian cancer cells.

**Fig. 2:**
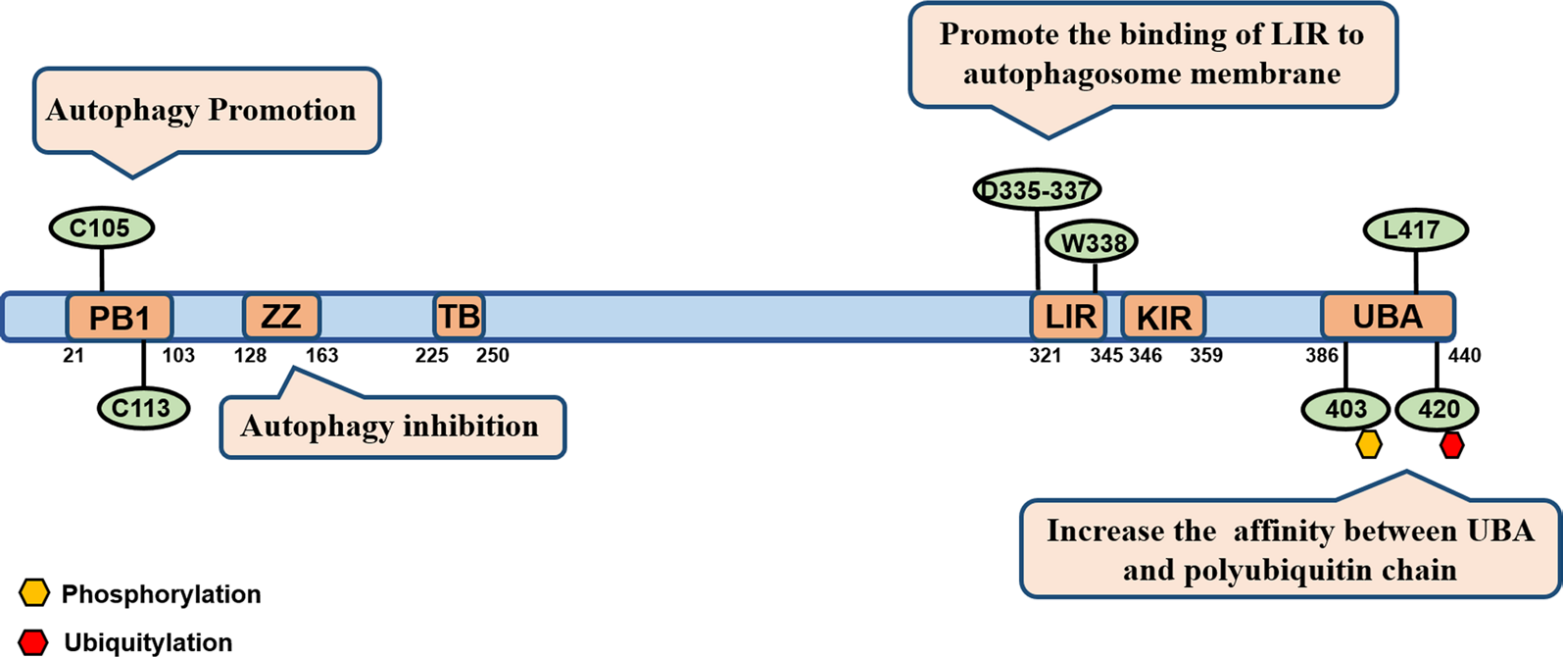
Schematic representation of p62 functional domains involved in autophagy regulation

Cha-Molstad et al. [32] found since ZZ domain may lock p62 in a close state, promoted the combination of ZZ domain and Nt-Arg leading to autophagy upregulation. Our study suggested that the LIR and UBA domains in p62 may modulate autophagic flux. We transfected SKOV3 cells with a vector encoding the L417V mutant (UBA mutant) that lost binding to ubiquitinated proteins and found increased autophagolysosomes in the UBA mutant-expressing cells, suggesting upregulated autophagic flux [33]. MTT assays revealed that UBA mutant-expressing cells showed reduced sensitivity to cisplatin compared with parental cells. Additionally, post-translational modifications of p62 also influence autophagy levels. Keap1/Cullin3 increased the co-localization of p62 and LC3 by ubiquitinating p62 at lysine 420 in the UBA domain and promotes the autophagy degradation pathway [34]. CK2 (casein kinase 2) increased the affinity of p62 to ubiquitin through phosphorylation of serine 403 in the UBA domain and promotes the clearance of ubiquitinated proteins by autophagy [26]. Specific mutations in the PB1 domain (C105A and C113A mutations) also significantly inhibited autophagy [35]. The effective mutations and post-translational modification sites reported to affect autophagy were shown in Fig. 2. Therefore, developing small molecule drugs that inhibit autophagy by targeting p62 may provide new strategies for combination treatment with cisplatin in ovarian cancer.

#### Mitophagy

Mitochondrial autophagy (mitophagy) maintains mitochondria mass and function by clearing damaged or overloaded mitochondria. Kingnate et al. [36] proposed that increased mitochondrial fusion and reduced mitochondrial fission may be one of the mechanisms of cisplatin resistance in ovarian cancer cells. Williams et al. [37] suggested that inhibition of mitochondrial fission leads to mitophagy suppression. The PINK1 (PTEN-induced putative kinase protein 1)/Parkin pathway is one of the regulators of mitophagy. The PINK1 serine/threonine kinase enters the mitochondrial membrane space through the translocase outer membrane complex. In healthy cells, the mitochondrial intramembrane rhomboid protease PARL mediates cleavage and inactivation of PINK1. However, in response to abnormal mitochondrial membrane potential, PINK1 accumulates in the mitochondrial outer membrane and recruits the E3 ubiquitin ligase Parkin to initiate mitophagy [38, 39].

Recent studies have indicated that p62 is not only found in the outer mitochondrial membrane but also localized to the inner mitochondrial membrane, which may be involved in mitochondrial function maintenance and morphology regulation [40]. VDAC (voltage-dependent anion channel), an important component of the mitochondrial permeability transition pore (mPTP), is a cysteine-containing protein that binds cisplatin [41]. VDAC maintains an open mPTP at low holding potentials but shows anion-selectivity upon increase of holding potential, which is associated with apoptosis regulation [42]. Geisler et al. [43] demonstrated that Parkin polyubiquitinates VDAC, and p62 recognizes ubiquitinated VDAC and mediates the degradation of damaged mitochondria. Whether p62 regulation of PINK and VDAC is involved in the cisplatin resistance of ovarian cancer cells is unknown and should be examined in future studies.

### p62 participates in pro-survival signaling regulation induced by cisplatin treatment in ovarian cancer

#### NF-κB signaling

NF-κB is one of the classical pro-survival signaling factors in cells. Phosphorylation of IκB kinase (IKK) promotes degradation of the NF-κB inhibitor IκB through the proteasome pathway, which subsequently activates NF-κB signaling. Many studies showed that the activated NF-κB pathway functions in promoting cell survival in ovarian cancer. Yang et al. [44] found that the E3 ligase TRIM52 (the tripartite motif 52) increased the expression of IKKβ and IKBα and promoted NF-κB subunit p65 nuclear translocation to activate the transcription of downstream MAPK9 (mitogen-activated protein kinase 9), BCL2 (B-cell lymphoma 2), CXCL8 (C-X-C motif chemokine ligand 8) and TNF (tumor necrosis factor) genes in SKOV3 and Caov3 ovarian cancer cells. Mabuchi et al. found that chemotherapy increased the levels of p-IκB and NF-κB transcriptional activity to higher levels in Caov-3 cisplatin-resistant ovarian cancer cells compared with A2780 cells. Furthermore, inhibiting NF-κB activity by *BAY 11*-*7085* enhanced the sensitivity of Caov-3 cells to cisplatin [45, 46]. These studies indicate that NF-κB may be involved in the mechanism of cisplatin resistance in ovarian cancer.

Recent studies found that p62 inhibition significantly suppressed activation of the NF-κB pathway [47]. We found that NF-κB signaling was activated in SKOV3/DDP cisplatin-resistant ovarian cancer cells. Inhibition of p62 by RNAi significantly reduced the translocation of p50/p65 into the nucleus and inhibited the transcriptional activity of NF-κB, which suggests that p62 may control cisplatin resistance in ovarian cancer cells by regulating NF-κB signaling [31]. Recent studies showed that TRAF6 and receptor-interacting protein 1 (RIP1) are both involved in p62-related NF-κB activation.

The TRAF6/NF-κB pathway was originally identified in immune cells [48]. The E3 ubiquitin ligase TRAF6 enhances the ubiquitination of IKKβ and the phosphorylation and degradation of IκB, leading to increased DNA-binding of NF-κB [49]. Moscat et al. demonstrated that RAS promoted the transcription of p62 through the ERK and PI3K pathways, which increase the oligomerization and polyubiquitination of TRAF6 and expressions of IKKα and IKKβ, the major upstream activators of the NF-κB pathway [17, 50, 51]. Another study showed that the p62 TB domain specifically binds to the TRAF domain in TRAF6, which results in autoubiquitination of TRAF6 [52]. Ubiquitination of TRAF6 was also inhibited in p62-knockout mice. Furthermore, a p62 UBA deletion mutant (F406V mutation) also inhibited the ubiquitination of TRAF6, which suggests that the C-terminal UBA domain of p62 is involved in TRAF6 regulation [53].

The RIP1 serine/threonine protein kinase is involved in the regulation of inflammatory signaling and various cell death pathways such as apoptosis and programmed necrosis [54, 55]. RIP1 function is tightly regulated by ubiquitination and deubiquitination. When cells are stimulated by tumor necrosis factor (TNF), TNF receptor 1 (TNFR1) forms trimers to recruit tumor necrosis factor receptor type 1-associated DEATH domain protein (TRADD), RIP1, E3 ubiquitin ligase TNFR-related factor 2 (TRAF2), cIAP1/2 (cellular inhibitor of apoptosis 1/2) as well as the linear ubiquitin chain assembly complex (LUBAC). RIP1 is rapidly polyubiquitinated with Lys63-linked and linear Met1-linked ubiquitin chains and activates TGFβ-activated kinase 1 (TAK1) and the IKK complex. Phosphorylated IκB is degraded by the ubiquitin–proteasome system, resulting in activation of NF-κB [56–58]. p62 directly binds RIP1 but not TRAF2, and the 117–439 residues in p62, which include the ZZ domain, are essential for binding to RIP1 [59]. We found that cisplatin activated the NF-κB pathway and increased K63-linked ubiquitination of RIP1 in SKOV3/DDP cells. Abolishing the regulation of RIP1 K63-linked ubiquitination by p62 increased the sensitivity of SKOV3 cells to cisplatin; deleting the ZZ domain (RIP1 interacting region) in p62 markedly decreased K63-linked ubiquitination of RIP1 in SKOV3 cells and inhibited NF-κB signaling activation [31]. These results suggested that the ZZ domain of p62 not only directly binds RIP1, but also participates in the regulation of RIP1 ubiquitination. NF-κB signaling regulates at least 400 genes encoding proteins involved in proliferation, apoptosis and inflammation [60]. When we blocked NF-κB signaling using p62 inhibition in ovarian cancer cells treated with cisplatin, the expressions of proliferation-related genes such as CCL2, IL6, TGFb and CSF3 genes were significantly suppressed and DNA synthesis was reduced [31].

#### Keap1/Nrf2 signaling

Mitochondria are the main location of cellular reactive oxygen species (ROS) production. Previous studies showed that cisplatin enters into cells and directly binds to mitochondria, leading to cytochrome C release, calcium-dependent mitochondrial swelling and production of ROS, which induces oxidative stress and reduces genomic stability [12, 61]. The antioxidant pathway and glutathione (GSH) are the main ways to clear ROS [62]. Current studies have shown that the Kelch-like ECH-associated protein 1 (Keap1)-nuclear factor erythroid 2-related factor 2 (Nrf2) pathway is one of the important antioxidant pathways. Under basal conditions, Keap1 interacts with Nrf2 and mediates its degradation. Exposure to electrophilic reagents (such as cisplatin) causes Keap1-Nrf2 complex disruption, leading to Nrf2 translocation into the nucleus to promote the expression of downstream antioxidant genes such as NQO1 and Hmox1 genes [63]. NRF2 also controls the gene transcription of several important enzymes involved in GSH synthesis and oxidation, maintaining the mitochondrial GSH pool [64]. The genes encoding ATP-binding cassette (ABC) transporters (known as efflux pumps) ABCC2 and ABCF2, which transport molecules across cellular membranes, are also target genes of NRF2 [65, 66]. Bao et al. [72] showed that Nrf2 knockdown inhibited the expression of ABCF2 and increased the sensitivity of ovarian cancer cells to cisplatin. Wu et al. [67] found that Nrf2 is highly expressed in A2780/DDP and COC1/DDP cisplatin-resistant ovarian cancer cells; inhibition of Nrf2 translocation into the nucleus significantly increased the gene expression of transferrin SLC401, a known iron exporter, which reversed the cisplatin resistance of ovarian cells caused by iron overload. These studies confirmed that the Keap1-Nrf2 pathway is involved in the cisplatin resistance mechanism of ovarian cancer cells. Some reports showed that Nrf2 interacts with the p62 promoter, which indicates that p62 may also be a target gene of Nrf2 [68]. Jena et al. [69] found that the E3 ubiquitin-protein ligase TRIM16 promotes p62-mediated autophagy degradation of ubiquitinated proteins by up-regulating Nrf2 under oxidative stress.

Recent studies have also confirmed that p62 is involved in regulation of the Keap1-Nrf2 pathway. Phosphorylation of p62 at serine 349 in the KIR domain increased its binding affinity to Keap1 and promoted Nrf2 activation [70]. Consistent with this data, other studies showed that Keap1/Cullin3 mediates p62 ubiquitination and increases the sequestration activity of Keap1, resulting in Nrf2 activation through non-canonical pathways [34]. We observed that SKOV3/DDP cells produced less ROS compared with SKOV3 cells upon cisplatin treatment, which suggests that cisplatin-resistant cells may have stronger antioxidant capacity. Furthermore, co-localization of p62 and Keap1 was observed in SKOV3/DDP cells and inhibition of p62 expression significantly attenuated the transcriptional activity of Nrf2 [71]. These results indicated that highly expressed p62 in SKOV3/DDP cells may protect ovarian cancer cells from oxidative damage caused by cisplatin by competing with Nrf2 for binding to Keap1.

**Fig. 3:**
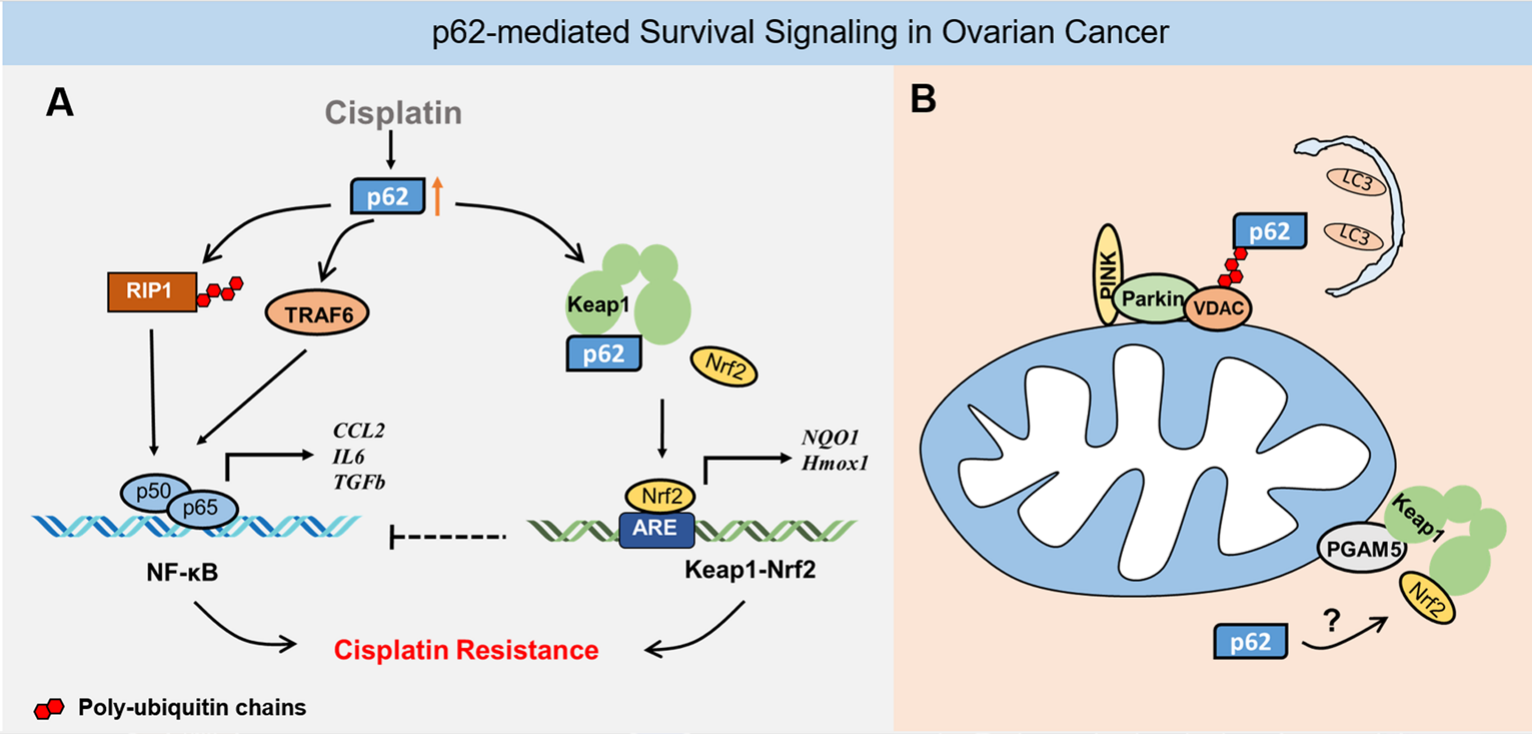
Pro-survival signaling regulation by p62 in ovarian cancer cells. **a** Highly expressed p62 activates NF-κB through RIP1 and TRAF6. p62 also competes with Nrf2 for binding to Keap1, which promotes the transcriptional activity of Nrf2. **b** p62 is recruited to function in PINK1/Parkin-mediated mitophagy; p62 may also be involved in regulation of the PGAM5-Keap1-Nrf2 complex, which is responsible for mitochondrial dynamics

Stępkowski et al. [72] showed that p62 plays an important role in apoptosis and autophagy by integrating the Keap1-Nrf2 and NF-κB signaling pathways. First, p62 promotes activation of the NF-κB signaling pathway; however, increased binding of p62 to Keap1 leads to release of Nrf2 from Keap1, which may affect the degradation of IKKβ by Keap1, resulting in NF-κB activation. This indicates a complicated role for p62 in pro-survival signal regulation. Additionally, PGAM5 (phosphoglycerate mutase family member 5), which is located in mitochondria and is a chaperone of Keap1, functions in apoptosis, programmed necrosis, and mitophagy. Mealey et al. [73] found that Keap1-Nrf2 interacted with PGAM5, and this complex contributed to mitochondrial retrograde trafficking. Furthermore, co-depleting p62 and Nrf2 inhibited mitochondrial clustering induced by the proteasome inhibitor *MG132*, which indicates that p62 may be involved in mitochondrial dynamics through Keap1-Nrf2 signaling regulation (Fig. 3). Together these findings indicate that investigating the role of p62 in the pro-survival signaling crosstalk may be a promising approach to develop strategies to overcome cisplatin resistance in ovarian cancer.

### Death signals recruited by p62 are involved in cisplatin resistance of ovarian cancer

Cisplatin causes cell death by activating apoptosis and programmed necrosis [74, 75]. Annunziata et al. found that ovarian cancer patients with tumors expressing low levels of the pro-apoptotic molecule caspase 8 showed shorter overall survival compared with those with high caspase 8 expression [76, 77]. Furthermore, another report demonstrated that p62 regulates caspase 8 activation induced by TNF [78]. Huang et al. [79] found that p62 accumulation caused by autophagy inhibitors *chloroquine* and *bortezomib* promoted the activity of caspase 8 in human colon carcinoma cells. Wang et al. [80] showed that caspase 8 activation may be regulated by intracellular death-inducing signaling complex (iDISC) on autophagosomal membranes. Furthermore, inhibition of ATG5 (autophagy-related gene 5) suppressed the formation of the autophagy membrane and reduced caspase 8 activation. Iurlaro proposed that persistent endoplasmic reticulum stress may promote the formation of iDISC, the autophagosome-associated platform, to activate caspase 8 [81]. Our group demonstrated that the combination of the autophagy inhibitor *chloroquine* and cisplatin significantly inhibited ovarian cancer growth in vivo, with accumulated p62 in tumor tissue and activated caspase 8. We previously found that deleting the p62 UBA domain, which is responsible for ubiquitin binding and self-oligomerization [82], inhibited cisplatin-induced caspase 8 activation, leading to chemoresistance in ovarian cancer cells. Further studies showed that the L417V mutation in the UBA domain, which reduced ubiquitin-binding activity, also suppressed caspase 8 activation and the co-localization with LC3 induced by cisplatin, which suggests that p62-mediated autophagy may participate in cisplatin resistance through caspase 8 regulation in ovarian cancer [33].

Cellular FLICE-inhibitory protein (cFLIP) is structurally similar to caspase 8 and forms a dimer with caspase 8 to inhibit its activity. cFLIP is highly expressed in ovarian cancer [83, 84]. Li et al. found that cFLIP knockdown significantly increased TRAIL-induced apoptosis in SKOV3 cells. Nazim et al. showed that increased autophagy flux inhibited the expression of cFLIP and enhanced TRAIL-induced apoptosis [85, 86]. Further studies are needed to clarify the role of cFLIP in p62-mediated caspase 8 activation in ovarian cancer with cisplatin treatment.

**Fig. 4:**
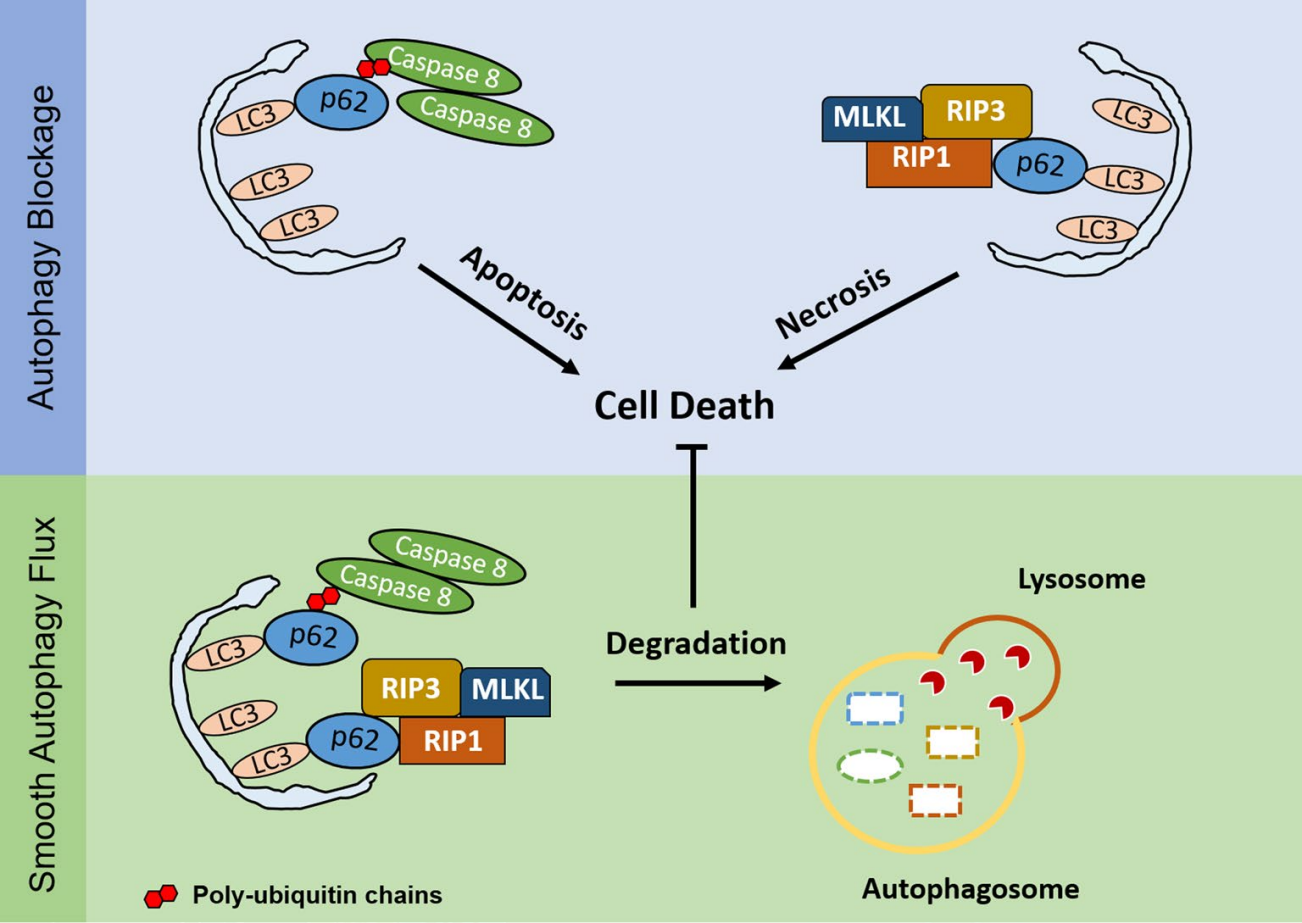
Death signals recruited by p62 in ovarian cancer cells. p62 induces apoptosis and programmed necrosis by recruiting pro-death partners on the autophagosome membrane while blocking autophagy flux

In the presence of RIP3, *cisplatin* induces formation of the necrosome containing the RIP1/RIP3/MLKL complex as core, which initiates programmed necrosis via ROS production [87]. Liu et al. [88] showed that the accumulation of p62 caused by autophagy inhibition promoted the formation of necrosomes. A recent study indicated that p62 induced programmed necrosis by recruiting RIP1 to assemble RIP3/MLKL on the autophagosome membrane in mouse prostate cells lacking Map3k7 [89]. These results suggest that p62 also works as a switch to determine the transition between apoptosis and necrosis. Targeting p62 to promote necrosis may be an alternative therapeutic strategy in ovarian cancers resistant to cisplatin-mediated apoptosis (Fig. 4).

## Conclusion and future perspectives

While early studies suggested that DNA was the main target of cisplatin [90], later reports demonstrated that cisplatin also triggers multiple changes in signals involved in proliferation, apoptosis and anti-oxidation by binding to macromolecular proteins and organelles [12]. In this review, we summarize our current understanding about the role of p62 in the mechanisms of cisplatin resistance in ovarian cancer cells.

p62 functions as a key receptor for autophagy. Increasing studies have demonstrated that autophagy is involved in chemotherapy resistance and several compounds have been identified that regulate autophagy in ovarian cancer (Table 2). We suggest that highly expressed p62 in ovarian cancer cells not only mediates selective autophagy to degrade excessive accumulated ubiquitinated proteins, but also exerts functions beyond autophagy. p62 also activates NF-κB by enhancing K63-linked ubiquitination of RIP1, promotes Nrf2 nucleus translocation to counteract oxidative damage caused by cisplatin by interacting with Keap1 and initiates cell death signals from autophagy flux blockage. These functions may explain why an autophagy inhibitor increased the efficacy of cisplatin in ovarian cancer cells. Together these studies indicate that p62 is involved in cisplatin resistant mechanisms by operating as a signal hub that regulates critical proteins in key signaling pathways that determine cell survival and death (Table 3).

**Table 2:** In vivo studies of compounds that regulate autophagy in ovarian cancer

Compounds	Effects on autophagy	Involved mechanism	Cell effects	Tumor model	References
*Paeonol*	Activator	Inhibiting mTOR/AKT	Protective	Xenograft (A2780 cells)	[91]
*JS*-*K*	Activator	Production of ROS/RNS	Cytotoxic	Xenograft (SKOV3 cells)	[92]
*Matrine*	Activator	Inhibiting mTOR/AKT	Cytotoxic	Xenograft (A2780 cells)	[93]
*Ormeloxifene*	Activator	Promoting ER stress and unfolded protein response	Cytotoxic	Xenograft (PA-1cells)	[94]
*Phyllanthusmin*	Inhibitor	Disruption of lysosomal function	Cytotoxic	Xenograft (OVCAR8-RFP cells)	[95]
*APG*-*1387*	Activator	Not clear	Cytotoxic	Xenograft (SKOV3 cells)	[96]
*Bortezomib*	Inhibitor	Promotion of ERK phosphorylation to suppress cathepsin B	Cytotoxic	Xenograft (MOSEC/LUC)	[97]

**Table 3:** Overview of p62-interacting proteins and associated signaling pathways that determine cancer cell fate

Domain	Partner molecules	Signaling	Cell survival/death	References
ZZ	RIP1	NF-κB	Survival	[31]
TB	TRAF6	NF-κB	Survival	[52]
KIR	Keap1	Keap1-Nrf2	Survival	[70]
UBA	Caspase 8	Autophagy	Death	[33, 78]
	VDAC	Mitophagy	Survival	[43]
168–224	EGFR	ERFR/Autophagy	Death	[98]

Notably, clarifying how p62 functions in chemotherapy may provide benefits for ovarian cancer clinical diagnosis and prognosis. Most ovarian cancer patients treated with cisplatin eventually develop resistance and show poor outcome. Iwadate examined p62 expression in tumor tissues of 266 patients with primary ovarian cancer and found that patients with high expression of p62 had poor prognosis [99]. Another study found that ovarian cancer patients with high expression of p62 had longer survival [100]. This paradox indicates that the prognosis of ovarian cancer patients may not be easily defined by p62 expression alone. Our study showed that patients with high expression of p62 and caspase 8 had longer survival and were negatively correlated with tumor-node-metastasis stage and relapse risk compared with patients with high p62 and low caspase 8 expression [33]. Considering the role of p62 in caspase 8 activation mentioned above, these findings suggest that patients with both overexpressed p62 and caspase 8 may be more sensitive to platinum-based chemotherapy; autophagy inhibitors may increase the sensitivity to cisplatin in patients with high p62 expression but low caspase 8 expression. These findings suggest that evaluation of p62 and its effector molecules could increase the accuracy of prognosis and optimize therapeutic strategies in ovarian cancer.

Several questions remain to be answered. For example, the role of accumulated p62 caused by autophagy blockage in NF-κB or Keap-1/Nrf2 signaling activation is unclear. Furthermore, the function of accumulated p62 induced by increasing transcription or degradation suppression is also unknown. Future studies should focus on alterations in the binding partners of p62 and the post-translational modifications that enhance p62 functions in ovarian cancer cells with cisplatin treatment. Together these findings may provide new strategies to overcome cisplatin resistance in ovarian cancer by targeting p62.

## Data Availability

Not applicable

## Abbreviations

ABC transporters: ATP-binding cassette transporters
ABCC2: ATP binding cassette subfamily C member 2
ABCF2: ATP binding cassette subfamily F member 2
aPKC: Atypical kinase
ATG5: Autophagy-related gene 5
BCL2: B cell lymphoma 2
CCL2: CC-chemokine Ligand 2
cFLIP: Cellular FLICE-inhibitory protein
cIAP: Cellular inhibitor of apoptosis
Cisplatin: *Cis*-diamminedichloroplatinum
CK2: Casein kinase 2
CXCL8: C-X-C motif chemokine ligand 8
DISC: Death-inducing signaling complex
DR: Death receptor
GSH: Glutathione
iDISC: Intracellular death-inducing signaling complex
IKK: IκB kinase
IL6: Interleukin 6
Keap1: Kelch-like ECH-associated protein 1
KIR: Keap1-interacting region
LIR: LC3-interacting region
MAP3K7: Mitogen-activated protein kinase kinase kinase 7
MAPK9: Mitogen-activated protein kinase 9
MLKL: Mixed lineage kinase domain-like protein
mPTP: Mitochondrial permeability transition pore
NQO1: NAD (P) H quinone dehydrogenase 1
NRF2: Nuclear factor erythroid 2-related factor 2
PINK1: PTEN-induced putative kinase protein 1
RIP1: Receptor-interacting protein 1
SLC401: Solute carrier 401
TAK1: TGFβ-activated kinase 1
TGF: Transforming growth factor
TNF: Tumor necrosis factor
TOM: Translocase outer membrane
TRADD: Tumor necrosis factor receptor type 1-associated DEATH domain protein
TRAF6: TNF receptor associated factor 6
TRIM52: The tripartite motif 52
UBA: Ubiquitin-associated
VDAC: Voltage-dependent anion channels
ZZ: Zinc finger
